# Immune age and biological age as determinants of vaccine responsiveness among elderly populations: the Human Immunomics Initiative research program

**DOI:** 10.1007/s10654-021-00767-z

**Published:** 2021-06-12

**Authors:** Jaap Goudsmit, Anita Huiberdina Johanna van den Biggelaar, Wouter Koudstaal, Albert Hofman, Wayne Chester Koff, Theodore Schenkelberg, Galit Alter, Michael Joseph Mina, Julia Wei Wu

**Affiliations:** 1 Department of Epidemiology, Harvard T.H. Chan School of Public Health, Boston, MA USA; 2 Department of Immunology and Infectious Diseases, Harvard T.H. Chan School of Public Health, Boston, MA USA; 3 Human Vaccines Project, New York, NY USA; 4 Ragon Institute of MGH, MIT and Harvard, Cambridge, MA USA; 5 Department of Pathology, Brigham and Women’s Hospital, Harvard Medical School, Boston, USA

**Keywords:** Immune aging, Biological age, Aging-related diseases, Vaccine responsiveness

## Abstract

The Human Immunomics Initiative (HII), a joint project between the Harvard T.H. Chan School of Public Health and the Human Vaccines Project (HVP), focuses on studying immunity and the predictability of immuneresponsiveness to vaccines in aging populations. This paper describes the hypotheses and methodological approaches of this new collaborative initiative. Central to our thinking is the idea that predictors of age-related non-communicable diseases are the same as predictors for infectious diseases like COVID-19 and influenza. Fundamental to our approach is to differentiate between chronological, biological and immune age, and to use existing large-scale population cohorts. The latter provide well-typed phenotypic data on individuals’ health status over time, readouts of routine clinical biochemical biomarkers to determine biological age, and bio-banked plasma samples to deep phenotype humoral immune responses as biomarkers of immune age. The first phase of the program involves 1. the exploration of biological age, humoral biomarkers of immune age, and genetics in a large multigenerational cohort, and 2. the subsequent development of models of immunity in relation to health status in a second, prospective cohort of an aging population. In the second phase, vaccine responses and efficacy of licensed COVID-19 vaccines in the presence and absence of influenza-, pneumococcal- and pertussis vaccines routinely offered to elderly, will be studied in older aged participants of prospective population-based cohorts in different geographical locations who will be selected for representing distinct biological and immune ages. The HII research program is aimed at relating vaccine responsiveness to biological and immune age, and identifying aging-related pathways crucial to enhance vaccine effectiveness in aging populations.

## Introduction

Infectious diseases present a major threat to the health of elderly populations. Compared to younger populations, older adults are at greater risk of developing severe symptoms, requiring hospitalization, and dying from infections. This is most evident in the current COVID-19 pandemic, where age is a strong predictor of the severity and outcome of SARS-CoV-2 infections, with adults aged over 65 years representing 80% of COVID-19 hospitalizations and having a more than 20-fold greater risk to die of COVID-19 [1, 2]. But also for other respiratory infections, such as influenza [3, 4], pneumococcal pneumonia [3, 5], and pertussis [6, 7], and other viral and bacterial infections including bacteraemia [8, 9] and severe gastroenteritis, risks exponentially increase above the age of 65 [10, 11].

**Fig. 1:**
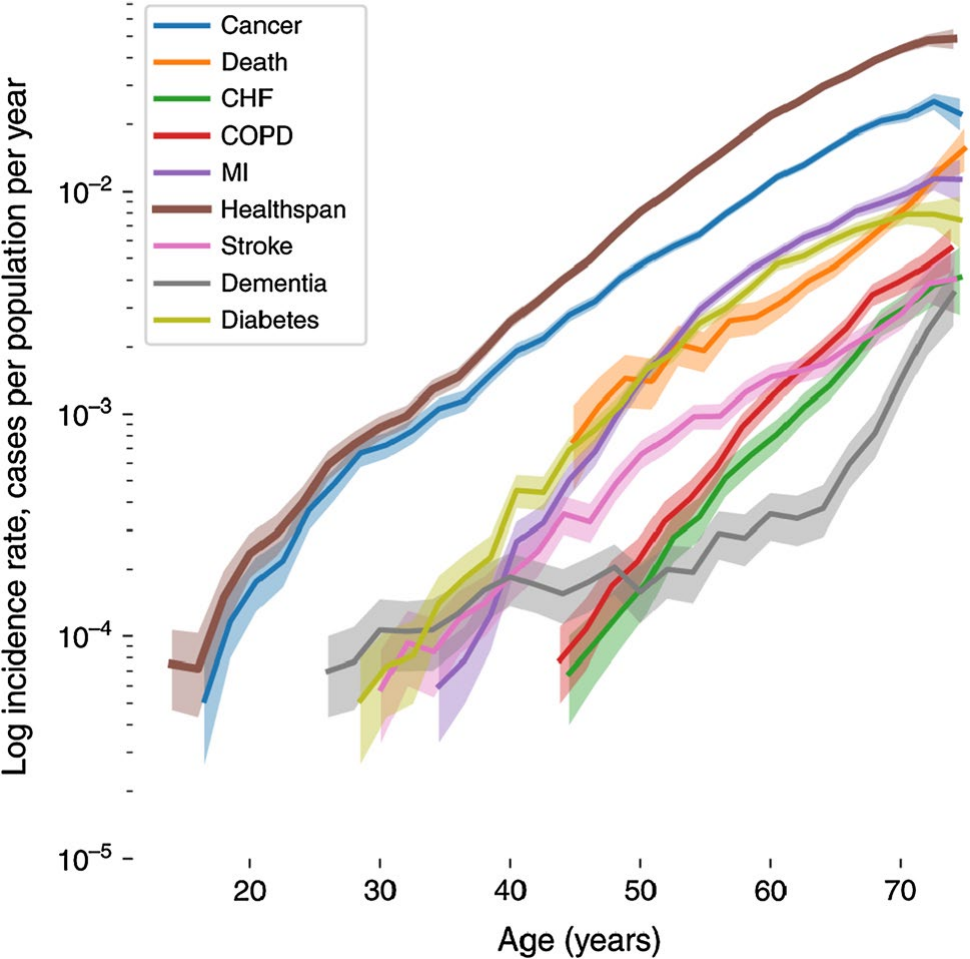
Incidence rates of the most prevalent chronic diseases, death, and healthspan based on clinical histories for over 300,000 people, aged 37 to 73 years old, participating in the UK Biobank cohort. Incidence rates for different chronic diseases, healthspan, and death increase at comparable, approximately exponentially rates with age. Disease incidence rates were calculated independently, with participants who develop more than one condition during the follow-up period counting for every disease they have. Healthspan was defined based in the first illness event occurrings. Shaded areas represent 95% confidence intervals. The graph was reproduced from Zenin et al., *Identification of 12 genetic loci associated with human healthspan. Commun Biol. 2019 Jan 30;2:41*

Main factors contributing to the increased vulnerability at older age include higher exposure, aging of the immune system, and co-morbidities [12–15]. Higher exposure primarily occurs through increased utilization of healthcare and long-term care facilities, where the risk of transmission and infection are high [16, 17]. Also the risk of re-activation of dormant infections such as herpes zoster [18] and tuberculosis [19] increases with age. The latter clearly signifies a failure of the aging immune system to control infections as a consequence of immunosenescence, i.e. the gradual deterioration of the immune system with natural age advancement. Another consequence of immune aging is a reduced ability to elicit effective immune responses to vaccines, as has been demonstrated for pneumococcal polysaccharide vaccines [20, 21], seasonal influenza vaccine [8], and herpes zoster vaccine [22]. Reduced vaccine responsiveness further limit the potential to protect older adults against severe infectious diseases. Finally, older adults developing severe infections often suffer from one or more non-communicable diseases, such as hypertension, ischemic heart disease, stroke, diabetes mellitus, chronic kidney disease, autoimmune, and neurodegenerative diseases: conditions that all increase exponentially at older age, as shown in Fig. 1 for UK Biobank participants [23].

The risks of developing non-communicable and communicable diseases with increasing age are not independent of each other: as clearly shown for COVID-19, pre-existing illnesses such as diabetes and hypertension are independent risk factors for developing and dying of a severe infection [24]. Both age-related non-communicable diseases and severe infectious diseases are associated with a chronic state of low-grade inflammation [25], which presents an important feature of the aging immune system known as ‘inflammaging'.

The ‘immunome’ is defined as the detailed map of immune reactions of a given host interacting with a foreign antigen, and ‘immunomics’ as the study of immunomes [26, 27]. In this paper we describe the objectives, hypotheses and methodological approaches of The Human Immunomics Initiative (HII), a joint project between the Harvard T.H. Chan School of Public Health and the Human Vaccines Project (HVP). The aim of HII is to decode the mechanisms and rules of effective immunity in aging populations through the exploration in large-scale population cohorts of biomarkers of immunosenescence, inflammaging, and risks for infectious and non-infectious diseases, and ultimately vaccine responses, in relationship to age. The differentiation between chronological and biological age is key in this process. Given the role of the immune system in age-related non-communicable diseases, HII takes the view that immunomics should not be limited to immune reactions with *foreign* antigens, but include also immune reactions to (modified) *self* antigens.

## Biological age as a predictor of all-cause mortality and age-related non-communicable diseases

Aging is characterized by a progressive loss of intrinsic capacity and functional ability, and increased risk for morbidities and death. Chronological age is an important predictor of morbidity and mortality but cannot account for heterogeneity in the decline of physiological function and health with advancing age. The realization that the rate at which people age is not universal, led to the concept that people have a biological age that reflects an individual’s global physiological status and functioning, his/her susceptibility to death and disease, and as such is a better predictor of lifespan and health span than chronological age [28–32].

**Fig. 2:**
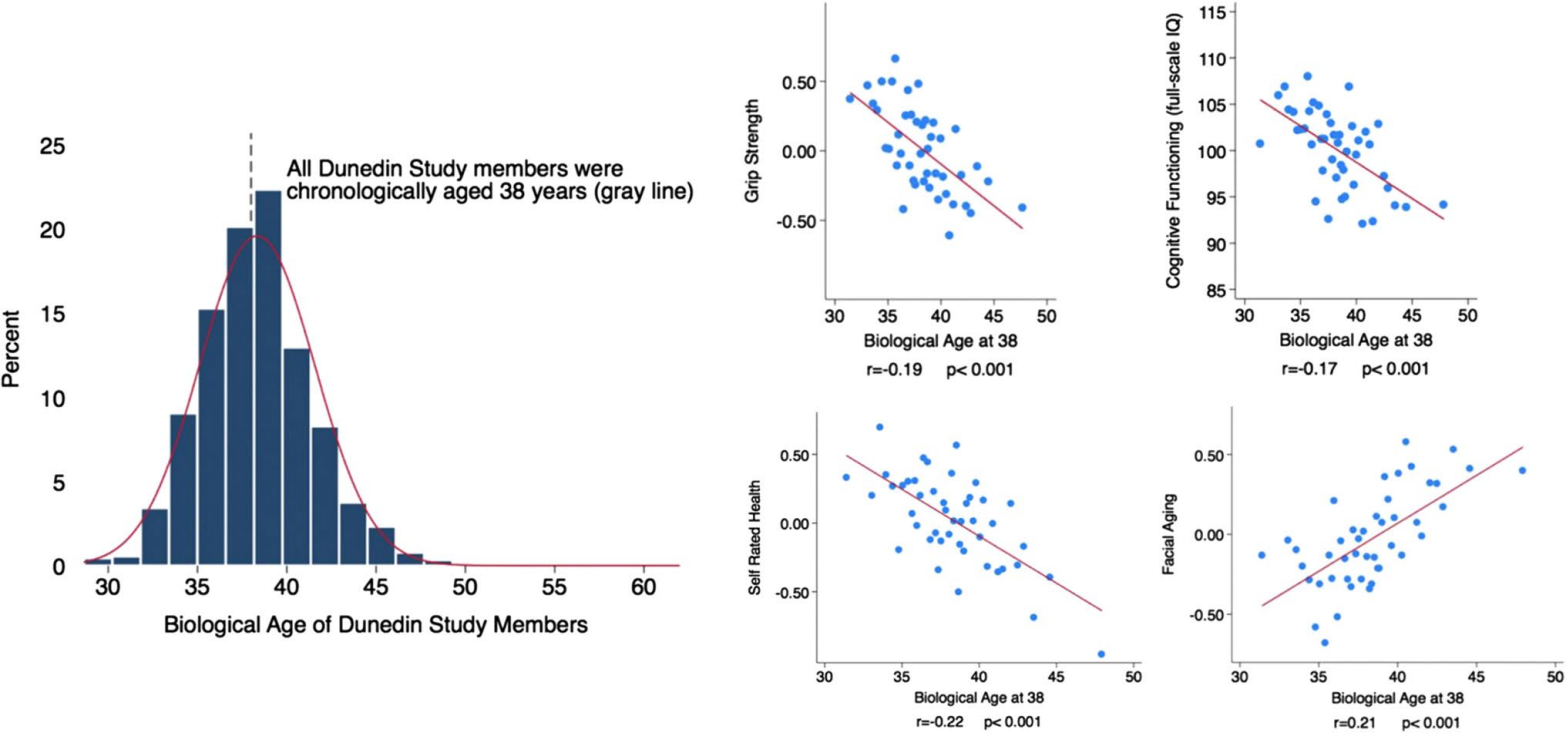
Biological versus chronological age in the Dunedin Study including 1037 young adults followed from birth to age 38 years. Biological age is normally distributed in a cohort of adults aged 38 years (left). Healthy adults who were aging faster exhibited deficits in physical functioning, showed evidence of cognitive decline, felt less healthy and were rated as looking older by independent observers (right). The figure shows binned scatter plots of the associations of biological age with grip strength, cognitive functioning, self-rated health and with facial aging. Each plotted dot point shows the mean for bins of data from *N* = 20 Dunedin Study members. Effect size and regression line were calculated from the raw data. Adapted with permission from Belsky WD et al., Quantification of biological aging in young adults. Proc Natl Acad Sci USA. 2015 Jul 28;112(30):E4104-10

In recent years, many studies have invested in studying biomarkers defining and predicting biological age, or hallmarks of aging [28, 33, 34]. Many of these studies have focused on biomarkers presenting a measure of biological age that is predictive of all-cause mortality. This includes physiological and biochemical biomarkers, such as for example the study of Levine et al. that used ten biomarkers: i.e. C-reactive protein, serum creatinine, glycated hemoglobin, systolic blood pressure, serum albumin, total cholesterol, cytomegalovirus optical density, serum alkaline phosphatase, forced expiratory volume, and serum urea nitrogen [28]. Other examples of biomarkers are systolic blood pressure, pulmonary vital capacity, creatinine, fasting glucose, as well as a Modified Mini-Mental Status Examination score presenting a ‘Healthy Aging Score’[33], molecular or epigenetic markers such as telomere length [35] and DNA methylation [29], or metabolomic predictors [36]. Biological age should, however, also account for differences in the physiological status and risk for age-related diseases among individuals of the same chronological age. More importantly, measures of biological aging based on clinically observable data, as opposed to those using molecular measures such as epigenetic clocks and leukocyte telomere length, tend to better capture risks for death and diseases and to be more robust predictors of aging-related outcomes [29]. By studying clinical measures representative of the physiological status of multiple organ systems (e.g. pulmonary, periodontal, cardiovascular, renal, hepatic, and immune function) repeatedly over a period of 12 years in middle-aged adults (the Dunedin Study), Belsky et al. [30] showed that already at midlife, before the onset of age-related diseases, an individual’s chronological age and biological age are divergent measures, with those who age more rapidly (i.e. who have an older biological age) being physically less able, showing cognitive decline and brain aging, self-reporting worse health, and looking older (Fig. 2).

Levine et al. [29] showed that biological age can differentiate between morbidity and mortality risks among chronological same-aged individuals. They further showed that using nine multi-system clinical chemistry biomarkers (albumin, creatinine, glucose, C-reactive protein, lymphocyte percent, mean cell volume, red blood cell distribution width, alkaline phosphatase, and white blood cell count), the difference between an individual’s biological versus chronological age is highly predictive of mortality, although the size of this effect decreases with age [37]. Wu et al. recently showed that physiological composite score-based biological age and its deviation from chronological age capture risks of death and all major aging-related morbidity, including dementia (manuscript submitted). Basic to the definition of biological age in the HII program is the risk-capturing summary of routine multi-system clinical chemistry biomarkers as initially introduced by Levine that are generally available for large scale population cohorts. Because of its robustness in predicting aging-related outcomes, we consider Wu’s physiological composite score-based biological age and its deviation from chronological age a better calibration tool for studying mechanistic aging than chronological age.

## Biological age as predictor of infectious diseases

As mentioned before, biological aging has been shown to be a stronger predictor for all-cause mortality and mortality from non-communicable diseases than chronological aging. However, it was not until the current COVID-19 pandemic that associations between biological aging and the risk of mortality from infectious diseases were studied. Since COVID-19 emerged, it has become clear that people of older age, and in particular those with co-morbidities, are at higher risk to develop and die of severe COVID-19 [1, 2, 24]. Applying the previously validated measure of biological age that was based on nine routine clinical biochemical markers, Levine and colleagues demonstrated that faster biological aging is associated with COVID-19 severity [38]. Importantly, Levine et al. did not determine biological age in people at the time they were diagnosed with COVID-19, but assessed their biological age 10-to-14 years prior to the onset of COVID-19. This was possible because the COVID-19 patients were previous participants of a large community cohort including over 500,000 subjects recruited between the ages of 40 and 70 during 2006 to 2010 in the United Kingdom (UK Biobank), and had routine clinical biomarkers determined at the time of recruitment. The observation that the deviation of biological age from chronological age is a strong predictor for developing a severe infectious illness a decade or more later, is novel and supports the notion that accelerated biological aging is a strong predictor of severe disease at older age, including infectious diseases.

## Immune age

Ahadi et al. [39] described four ‘ageotypes’, or biological pathways of aging, including: immunity, metabolic pathways, liver dysregulation, and kidney dysregulation. For each of these pathways, people may age at different rates. Like for other systems, changes in immune functioning over lifetime are likely less dictated by chronological age than by individual trajectories that may be influenced by genetics, epigenetics, and environmental factors [40–42]. ‘Immune age’ may therefore be a predictor of infectious and non-communicable diseases and vaccine responsiveness at older age.

The human immune system involves more than 1500 genes/proteins in many interconnected pathways and processes [43]. The last decade has seen an explosion in high-throughput technologies that allow us to study the human immune system in small volumes of blood based on the detection of multi-level changes in molecular immune pathways and networks. This includes but is not limited to platforms for RNA-sequencing, flowcytometry, and high-performance liquid chromatography or mass spectometry for plasma proteomics and antibody glycosylation [44, 45]. Consequently, a holistic study of the immune system in relation to individual and population health and disease is now technically possible, and with that the opportunity to phenotype Immune Age. Pulendran and colleagues [22] used this approach to explore immune pathways in response to Herpes Zoster vaccination in both younger and older adults, and identified immune and metabolic correlates predictive of differences in vaccine immunity in older vs younger adults [22]. Another example is a study by Alpert et al. [46] who used an ‘omics’ approach applied to a longitudinal cohort of adults sampled multiple times over the course of nine years, to identify an immune age algorithm that was predictive of all-cause mortality. Pulendran and Alpert both used isolated immune cell populations to phenotype cell gene expression profiles (transcriptomics). Access to isolated blood immune cells, however, requires specifically designed, smaller-scale studies. Usually immune cells are not available from large community-based population cohorts. In contrast, plasma samples are typically bio-banked at larger quantities. High-throughput platforms to analyse functional properties of antibody responses are now available and have been used to study immunity in vaccinated or infected humans [47, 48].

Antibodies play an important role in both immunosenescence and inflammaging: two processes that both define immune aging [49]. As mentioned, immunosenescence is an age-related weakening of the immune system´s ability to respond to danger signals and clear pathogens. One aspect of immunosenescence is a narrowing of the B-cell repertoire against non-self-antigens, and a failure of self-tolerance mechanisms to deplete B-cells recognizing self-antigens. As a result, the spectrum of antibodies recognizing pathogens and danger signals becomes smaller, while the spectrum of auto-antibodies is thought to increase with aging [50, 51]. Inflammaging is an age-related development of a chronic state of low-grade inflammation that is caused by a sustained activation of innate immune cells. Inflammasomes may play an important role in inflammaging and age-related diseases [52, 53].

Inflammasomes are protein complexes that are formed when specific receptors of innate immune cells recognize microbial or danger signals. Inflammasomes were first discovered by the team of Tschopp et al. [54] and a more detailed description on inflammasomes and their function can be found in a review by Schroder and Tschopp. In brief, inflammasomes stimulate the production of the pro-inflammatory cytokines IL-1ß and IL-18, and lead to a pro-inflammatory type of cell death known as pyroptosis. The assumed primary role of inflammasomes is to mediate protection against invading pathogens, with pyroptosis leading to the death of infected cells and activation of adaptive immune responses to clear infection. A number of host mechanisms also suppress inflammasome activation, presumably in order to limit the extent of potentially dangerous immune activation. Aberrations in inflammasome-mediated signaling and control mechanisms may result in increased susceptibility for infections and/or more severe disease symptoms, and could predispose to developing autoreactive immune responses. This may involve antibodies that can potentiate or suppress the inflammasome [47, 55, 56].

Antibody functionality is in part regulated through glycan structures present on the antibody’s constant region. Hundreds of differentially glycosylated antibody variants can be present in an individual at any given time, some of which are associated with pro-inflammatory responses and others with anti-inflammatory properties. This large individual variability contributes to individual differences in function of the immune system. Aging, auto-immunity, non-communicable, and severe infectious diseases are all associated with immunoglobulin G (IgG) glycan species promoting inflammatory responses [47, 49, 57–59]. Glycans therefore may regulate the potentiating or suppressing effect of antibodies on innate immune responses. At the same time, antibody glycans may contribute to immune senescence as glycan species expressed at older age may be associated with lower receptor binding and affinity, and hence possibly reduced pathogen clearance [58, 60].

By analysing IgG glycosylation in relation to chronological age in a training set and in a subsequent validation set including more than 5000 individuals from four different European populations, Krištić et al. [61] developed a predictive model consisting of only three glycans, called ‘Glycan Age’: this model can predict chronological age with an error of 9.7 years, and explain nearly 60% of variation in chronological age and sex. In comparison, conservative age biomarkers such as telomere length accounted for as little as 15% to 25% of variance of age in this study. The “Glycan Age” index was also found to correlate with physiological markers that change with aging and possible predictors of biological age. This suggest that antibody glycosylation may be a predictor of biological age, and potent biomarker of immune age: two hypotheses that are fundamental and will be studied in the HII program.

Antibody glycosylation profiles that are progressively seen with aging are also associated with metabolic health [62]. Metabolic processes play both direct and indirect roles in inflammaging and age-related infectious and non-infectious diseases [36, 63, 64]. Immune aging, risk for severe infectious diseases, and metabolomic health therefore are likely interrelated, and may be mediated through similar changes in antibody glycosylation. This interconnectivity of immune aging and metabolic pathways has become evident in recent studies of severe COVID-19 outcomes in older patients [25, 65–70], where inflammasome activation [71] and altered antibody glycosylation [72, 73] have been associated with a reduced capacity of older individuals to clear the SARS-CoV-2 virus [74] and subsequent enhancement of hyper-inflammation responses [75]. Emerging observations that COVID-19 has the potential to enhance and accelerate processes of immunosenescence and inflammaging [76] is novel evidence that infectious diseases can negatively impact immune age. Hence, immune age may depend to a certain extent on infections that individuals have experienced in the past. Immunity and the ability to respond effectively to vaccination is thus shaped by a complex of interacting factors, including aging, immunosenescence, inflammaging, metabolomics and antibody glycosylation.

## The HII program

**Fig. 3:**
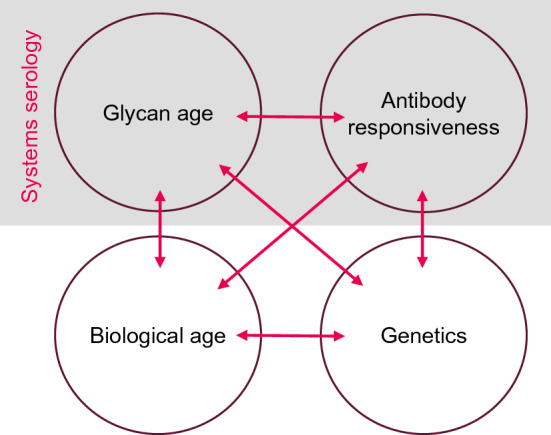
Set of interacting factors shaping the aging immune system studied by HII

In order to decode mechanisms and rules of effective immunity in aging populations, the Human Immunomics Initiative (HII) will leverage the strength of large prospective population cohorts that provide a wealth of information on the health status and clinical endpoints of a large number of participants who are representative for the general population. Given that bio-banks of population cohorts typically store plasma but not immune cells, HII will focus on high-throughput analyses that can be conducted using plasma to identify biomarkers of immune age and phenotype humoral immuneresponses. This variant of “systems serology” [77, 78] may include: phage-immunoprecipitation-sequencing (PhIP-Seq)-based methods for the comprehensive analysis of serum antibodies to human pathogens (“VirScan”) and self antigens (“peptidome”) developed by Stephen Elledge [79–83]; antibody glycosylation analysis as described above for Glycan age and in a further expanded approach, and additional analyses such as transcriptomics and metabolomics. Next to system serology, biological age as determined from routine clinical biochemical parameters will be used to characterize the background of the aging person. When available, genetics will also be considered, as genetic loci have been shown to be associated with healthspan [23] as well as with glycosylation of IgG [84], and thus immune age (Fig. 3).

The program will consist of two phases, with phase 1 aimed at exploring and validating the concept of immune age as based on systems serology parameters in relation to biological age and age-related illnesses. In the second phase, biological age and immune age will be studied as predictors of vaccine responsiveness in vaccine studies nested within large prospective population-based cohorts in different geographical locations worldwide.

**Fig. 4:**
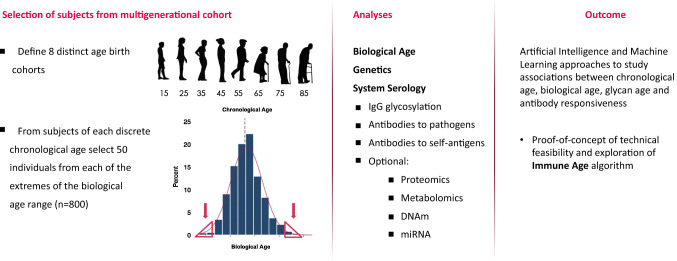
Design of a phase 1 technological feasibility study to explore the associations between chronological age, biological age and immune age as defined by glycan age and antibody responsiveness and genetics

The first step of phase 1 will explore the association of chronological age, genetics, biological age and immune age based on glycan age and antibody responsiveness in a large-scale prospective cohort study. The model of biological age as developed by Levine, and later further built upon by Wu using routine clinical biomarkers, will be applied to select from each age group (e.g. participants aged 15 ± 2 years; 25 ± 2 years; etc. up to 85 ± 2 years at the time of sampling) subjects who are at the extremes of biological age, i.e. who have the youngest or oldest biological age for their chronological age. Plasma samples from these subjects will then be analysed to define immune age based on antibody glycosylation analysis and deep serological profiling for non-self and self-antibodies (Fig. 4). This first phase is planned to be conducted in 2021.

**Fig. 5:**
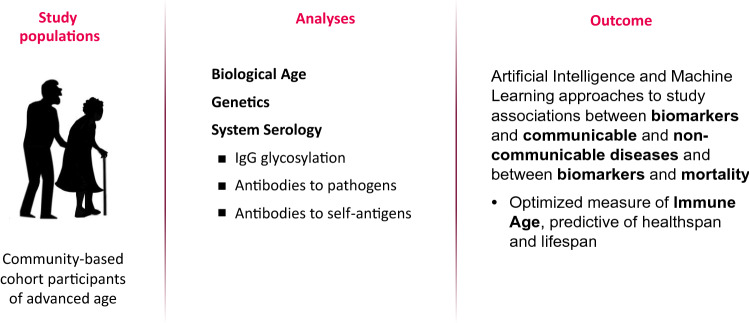
Design of phase 1 study to derive and validate an optimized measure of immune age that is predictive of healthspan and lifespan

In the second step of phase 1, the HII program aims to study the immune status at advanced age as characterized by biological age, glycan age and antibody responsiveness, as predictor of healthspan and life span in community-based cohorts of aged adults. Plasma samples will be analysed for immune age using the same technologies as in the first step in the program. Modelling of these B-cell immune biomarkers, together with biomarkers of biological age and genetic markers, against mortality and morbidity risk is expected to provide an optimised measure of immune age that is predictive of healthspan and lifespan (Fig. 5).

**Fig. 6:**
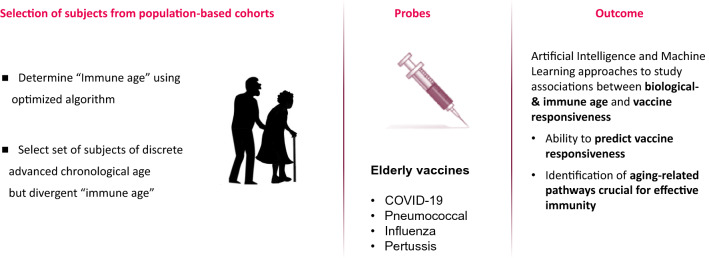
Design of phase 2 study to assess the predictive value of biological- and immune age algorithms for vaccine responsiveness and identification of pathways crucial for effective immunity in aging populations

In phase 2 of the HII program we will focus on translating the learnings of phase 1 on effective immunity at older age to a current real-life situation of protecting vulnerable aging population against infectious diseases, including COVID-19. Like phase 1, phase 2 of the program will be conducted nested within existing population cohorts. The same instruments and analytical assays as used and validated in phase 1 will be applied to determine biological age and immune age. The aim is not to conduct clinical vaccine trials. Instead, responses to for example COVID-19 vaccines will be studied when study subjects receive such a vaccine as part of national vaccination programs. This can also involve influenza-, pneumococcal- and pertussis vaccines that in many high-income countries are part of routine vaccination programs for elderly. The objective is to conduct this part of the program in different parts of the world, partnering with population cohorts in different geographical locations worldwide, including Europe, the US, and Asia. This means that we will not only study different populations but also different vaccines, as different vaccines are available in different parts of the world. From each cohort, 100–200 elderly of distinct biological and immune age will be selected. Tools that will be used to define biological and immune age will be as they are informed and optimized during Phase 1 of the program (Fig. 6).

In our proposed approach, we aim to first construct immune age composite scores based on all-cause mortality, and then evaluate their associations with risks of both non-communicable and communicable diseases including immune responsiveness to various vaccines. The resulting data will provide information regarding how consistent these associations will be. To study immunity and predictability of immune responsiveness to vaccines, immune parameters need to be considered as described in Sect. 4. As mentioned, our “Immune Age” algorithm will be based on a selection of biomarkers from the entirety of parameters measured in the described Systems Serology that can be assessed in samples available in large-scale population cohorts (to be able to derive and validate predictive algorithms). Standard regression models are limited in their prediction capacity, given the challenge to account for complex interactions and correlation involved with high-dimensional datasets (i.e., data collections with a multitude of variables). We therefore propose to apply artificial intelligence techniques, in particular cohort-based machine learning methods [85, 86], such as elastic net regression [87], random forest models [88], and recurrent neural networks [89], for their ability to account for the complex correlation structures between multi- system serological measures. The predictive accuracy of algorithms assumes rather complete input. Another important assumption is the sufficient sample size relative to the number of parameters assessed, to avoid data overfitting. K-fold cross-validation will be essential [85], to enhance the validation of robustness by using successive and mutually exclusive validation datasets.

The ultimate goal is to relate vaccine responsiveness as assessed by the clinical assays relevant for each of the different vaccines to biological and immune age, and identify crucial aging-related pathways that lead to underperformance and can potentially be adjusted, through interventions or vaccine optimization, to enhance vaccine effectiveness.

## Summary

In summary, by standardization of methods and techniques to profile humoral immune responses in well-characterized human cohorts, the Human Immunomics Initiative strives to identify pathways that are crucial to improve vaccine-induced protection in aging populations. The inclusion of geographically diverse populations, which will capture variation in a population’s immune status due to genetic and environmental differences, will strengthen the ability of the program to interrogate the aging human immune system and identify universal pathways of effective immunity. It is anticipated that knowledge acquired through this program can be translated directly to study outcomes for other vaccines against other serious infectious diseases that negative impact the health- and lifespan of elderly.
